# Supplementary material for: Impact of Histotripsy on Development of Intrahepatic Metastases in a Rodent Liver Tumor Model
Source: Cancers (Basel). 2022 Mar 22;14(7):1612. doi: 10.3390/cancers14071612 (PMC8996987; doi:10.3390/cancers14071612)
Supplement: Supplementary file 1 [file cancers-14-01612-s001.zip › cancers-1648056-supplementary.pdf]

Supplementary Materials

Impact of Histotripsy on Development of Intrahepatic Metastases in a Rodent Liver Tumor Model

Tejaswi Worlikar, Man Zhang, Anutosh Ganguly, Timothy Hall, Jiaqi Shi, Lili Zhao, Fred Lee, Mishal Mendiratta-Lala, Clifford S. Cho and Zhen Xu

Table S1. Tumor Response Summary.

Group	ID	Survival (Week)	Week 0 (mm^3)	Week 1 (mm^3)	Week 2 (mm^3)	Week 3 (mm^3)	Week 4 (mm^3)	Week 5 (mm^3)	Week 6 (mm^3)	Week 7 (mm^3)	Week 8 (mm^3)	Week 9 (mm^3)	Week 10 (mm^3)	Week 11 (mm^3)	Week 12 (mm^3)
Histotripsy	H1	12	40.27	17.55	0	0	0	0	0	0	0	0	0	0	0
	H2	12	157.50	66.40	3.55	0	0	0	0	0	0	0	0	0	0
	H3	12	48.92	1.05	0	0	0	0	0	0	0	0	0	0	0
	H4	12	161.23	60.20	6.00	0	0	0	0	0	0	0	0	0	0
	H5	12	150.90	102.0	81.64	0	0	0	0	0	0	0	0	0	0
	H6	12	120.41	38.68	0	0	0	0	0	0	0	0	0	0	0
	H7	6	50.63	111.3	71.11	97.04	302.57	2490	7967	-	-	-	-	-	-
	H8	6	42.91	127.5	175.08	2032	1329.8	1950	6487	-	-	-	-	-	-
	H9	12	57.61	57.27	20.93	0	0	0	0	0	0	0	0	0	0
	H10	7	51.28	34.16	2.198	0	0	0	0	0	-	-	-	-	-
	H11	7	67.95	37.68	0	0	0	0	0	0	-	-	-	-	-
Control	C1	3	73.85	1094	1441	2747	-	-	-	-	-	-	-	-	-
	C2	1	233.35	11528	-	-	-	-	-	-	-	-	-	-	-
	C3	1	164.06	4391	-	-	-	-	-	-	-	-	-	-	-
	C4	1	193.63	4729	-	-	-	-	-	-	-	-	-	-	-
	C5	1	67.25	1421	-	-	-	-	-	-	-	-	-	-	-
	C6	1	278.62	2555	-	-	-	-	-	-	-	-	-	-	-
	C7	1	156.71	8937	-	-	-	-	-	-	-	-	-	-	-
	C8	2	55.16	454	2905	-	-	-	-	-	-	-	-	-	-
	C9	2	64.52	3727	4391	-	-	-	-	-	-	-	-	-	-
	C10	2	28.88	587.5	9107	-	-	-	-	-	-	-	-	-	-
	C11	1	39.25	1198	-	-	-	-	-	-	-	-	-	-	-

Note: Tumor measurements are made on MRI images in 3 dimensions, which is used to estimate tumor burden. If no tumor is detected on MRI '0' is recorded. Week 0 is the histotripsy timepoint (7–9 days post inoculation). n = 2/11 histotripsy animals still surviving at 7 weeks with no observable tumor were euthanized due to COVID shutdown (ID H10 and H11).
